# Supplementary material for: Prevalences of Tobamovirus Contamination in Seed Lots of Tomato and Capsicum
Source: Viruses. 2023 Mar 30;15(4):883. doi: 10.3390/v15040883 (PMC10146847; doi:10.3390/v15040883)
Supplement: Supplementary file 1 [file viruses-15-00883-s001.zip › viruses-2237774-supplementary.pdf]

Dall et al 2023. Supplementary data for
'Prevalences of Tobamovirus Contamination in Seed Lots of Tomato and Capsicum'

Table S1. Ranked prevalence estimates for tobamoviruses in contaminated tomato and capsicum seed lots.

Ranking by prevalence	Virus identity	Host	Estimated prevalence (%)	Cumulative contamination percentile	# seed tested
1	PMMoV	Capsicum	0.3880	3.45	12,000
2	ToMV	Capsicum	0.2080	6.90	10,400
3	ToMV	Tomato	0.1080	10.35	12,000
4	ToMV	Capsicum	0.0891	13.80	12,800
5	ToMV	Tomato	0.0693	17.25	17,600
6	PMMoV	Capsicum	0.0655	20.70	11,600
7	ToMV	Capsicum	0.0600	24.15	18,000
8	PMMoV	Capsicum	0.0433	27.60	12,000
9	PMMoV	Tomato	0.0300	31.05	20,000
10	ToMV	Capsicum	0.0280	34.50	10,000
11	ToMV	Tomato	0.0180	37.95	10,000
12	PMMoV	Capsicum	0.0150	41.40	12,000
13	PMMoV	Capsicum	0.0140	44.85	20,000
14	ToMV	Capsicum	0.0140	48.30	20,000
15	TMV	Capsicum	0.0080	51.75	20,000
16	PMMoV	Capsicum	0.0080	55.20	20,000
17	ToMMV	Capsicum	0.0080	58.65	20,000
18	ToMV	Capsicum	0.0080	62.10	10,000
19	PMMoV	Capsicum	0.0080	65.55	20,000
20	ToMMV	Tomato	0.0080	69.00	20,000
21	ToMV	Tomato	0.0077	72.45	10,400
22	PMMoV	Capsicum	0.0067	75.90	12,000
23	PMMoV	Capsicum	0.0067	79.35	12,000
24	ToMV	Capsicum	0.0067	82.80	12,000
25	ToMMV	Tomato	0.0057	86.25	14,000
26	ToMV	Tomato	0.0046	89.70	17,600
27	ToMV	Capsicum	0.0040	93.15	20,000
28	ToMV	Tomato	0.0040	96.60	20,000
29	ToMV	Tomato	0.0040	100.05	20,000
